# Our Journal

**Published:** 1855-07

**Authors:** 


### OUR JOURNAL.

The next number will complete the 2d volume of our Journal, and we take this occasion to say that from the large increase to our subscribers, we are determined to progress, and from the encouragement we received at the last meeting of the Medical Society of the State, we believe we will soon be able to issue a monthly at the same rates and of the same size. All we want is an increased number of subscribers, and then it will be issued monthly. One hundred more pre-paying subscribers will do it after the payment of what is due us, and this number can be raised if our friends will work. We will give a premium to that individual who will obtain the greatest number for the next volume which shall be worthy of giving and accepting.

The individual who will forward ten dollars shall have six copies sent to his order, which will be one volume gratuitously to himself.

We earnestly hope our friends will make the effort and in order that they may see how favorably the work is received by the profession over the Union, we will copy and publish the editorials found in the other medical journals respecting its standing and usefulness.

